# Toxicity of Per- and Polyfluoroalkyl Substances to Nematodes

**DOI:** 10.3390/toxics11070593

**Published:** 2023-07-07

**Authors:** Tingting Ma, Xia Pan, Tiantian Wang, Xiuhua Li, Yongming Luo

**Affiliations:** 1 Wenzhou Key Laboratory of Soil Pollution Prevention and Control, Zhejiang Industry and Trade Vocation College, Wenzhou 325002, China; ttmaxiaotu@126.com; 2 College of Resource Environment and Tourism, Hubei University of Arts and Science, Xiangyang 441053, China; ttw0410@163.com; 3 CAS Key Laboratory of Soil Environment and Pollution Remediation, Institute of Soil Science, Chinese Academy of Sciences, Nanjing 210008, China; xhli@issas.ac.cn (X.L.); ymluo@yic.ac.cn (Y.L.)

**Keywords:** perfluorooctane sulfonate, soil fauna, *Caenorhabditis elegans*, ecotoxicity, substituted pollutants

## Abstract

Per- and polyfluoroalkyl substances (PFASs) are a class of compounds that persist in the environment globally. Besides being transported to the soil and sediments, which act as their sinks, PFASs can be transferred to several species of higher organisms directly or via bacteria, eliciting a wide range of adverse effects. *Caenorhabditis elegans* has been widely used in toxicological studies and life science research owing to its numerous advantages over traditional vertebrate models; notably, *C. elegans* has 65% conserved human-disease-associated genes and does not require ethical approvals for experimental use. This review covers a range of topics, from reported accumulation characteristics and lethal concentrations of PFAS in *C. elegans* to the mechanisms underlying the toxicity of PFAS at different levels, including reproductive, developmental, cellular, neurologic, oxidative, metabolic, immune, and endocrine toxicities. Additionally, the toxicity levels of some PFAS substitutes are summarized. Lastly, we discuss the toxicological mechanisms of these PFAS substitutes and the importance and promising potential of nematodes as in vivo models for life science research, epidemiological studies (obesity, aging, and Alzheimer’s disease research), and toxicological investigations of PFASs and other emerging pollutants compared with other soil animals or model organisms.

## 1. Introduction

Per- and polyfluoroalkyl substances (PFASs) have been used for more than half a century for industrial and commercial applications and have been studied at regional, national, and international scales owing to concerns regarding their adverse effects on ecology and human health. PFASs comprise a large class of persistent organic pollutants (POPs) ubiquitously detected in soil, water, and other environmental media. Additionally, they are found in different types of foods, plants, animals (fish, birds, and mammals), and human breast milk and blood. PFASs are characterized by a complex degradation process, long-distance migration, and biological accumulation [1,2]. The presence and persistence of these compounds result in their bioaccumulation and biomagnification at multiple levels within the natural food web, challenging the accurate assessment of ecological risks while accounting for the vast differences in species habitat and compound exposure [3,4]. Perfluorooctanesulfonic (PFOS) and perfluorooctanoic acids (PFOAs) are the most prominent chemical contaminants in this group of synthetic chemicals and are associated with various toxicities and disruptive effects on the immune, metabolic, neural, and endocrine systems of different living organisms [5,6].

Soil communities are extremely complex and diverse, ranging from microscopic bacteria and fungi to larger organisms, such as nematodes, mites, earthworms, ants, and moles [7]. Soil animals are invertebrates that spend a considerable period of their lives in the soil, exerting a certain influence on it. These animals are important for studying soil biological effects, soil health, ecosystem succession, and the degree of disturbance in a terrestrial ecosystem. Additionally, they are the most sensitive indicator species for analyzing changes in the soil environment [8,9]. Among the large functional groups of soil animals, nematodes occupy an important position at the center of the soil food web and have many trophic preferences and life strategies, affecting primary production, decomposition, energy flow, and nutrient cycling [10]. Furthermore, owing to their high abundance, omnipresence in most terrestrial and aquatic ecosystems, and good adaptation to an extensive range of environmental conditions, nematodes have become ideal model organisms for environmental and ecological risk assessments, particularly for determining contamination status [11,12].

Besides direct contact with different environmental matrices, PFASs are often transferred via bacteria to higher organisms, such as *Caenorhabditis elegans* [13]. *C. elegans* is a eukaryotic, multiorgan, transparent nematode that undergoes four larval stages (L1–L4) before reaching adulthood, with a total longevity of approximately 2–3 weeks at 20 °C. It lives in interstitial soil water and survives by feeding on microbes [14]. *C. elegans* is an internationally approved model organism with a small size (approximately 1 mm as an adult). Its simple structure, easy laboratory cultivation, low cost, simple experimental operation, short growth cycle, multiple offspring production, well-defined genetic background, and variable meiotic behavior give it inherent advantages in the study of germline cells [15]. Notably, genetic sequencing of *C. elegans* revealed that 60–80% of its genes are homologous to those of humans [14,15,16,17]. Furthermore, *C. elegans* is used as a sensitive bioindicator in various exposure media (soil, water, and sediment) used in toxicological studies to observe different toxicological endpoints, such as mortality, reproductive toxicity, and behavioral changes [18,19,20,21].

PFOS and PFOA are the two POPs most commonly detected at levels higher than those of other PFASs [22]. Compared with the long-chain PFASs, the environmental behavior and ecotoxicity of extensively employed short-chain PFAS alternatives, including perfluorobutanoic acid (PFBA), perfluorobutane sulfonate (PFBS), potassium salt of trifluorohexane-1-sulfonate (PFHS), perfluorohexanoic acid (PFHxA), and a precursor substance, N-ethyl perfluorooctyl sulfonamide ethanol (N-EtFOSE), have become increasingly relevant [23,24]. Long before PFOS and its salts were included as POPs under the Stockholm Convention of 2009, PFBS was launched by the 3M company in 2003 as a low-toxicity and low-enrichment substitute for PFOS to reduce its negative influence on the ecological environment and human health [25,26]. Furthermore, hexafluoropropylene oxide dimer acid, also known as GenX, is an emerging PFAS developed to replace PFOA and is relatively resistant to abiotic and biotic degradation [27]. In this review, we report the accumulation characteristics, acute toxicity (mainly lethal effect concentrations), and chronic toxicity of PFASs to *C. elegans* (Table S1), including effects on its cellular structure and reproductive, developmental, nervous, oxidative, metabolic, immune, and endocrine systems. Additionally, we describe the toxicological mechanisms of different PFASs and some PFAS substitutes at various toxicity levels. Lastly, we discuss the application of nematodes in future epidemiological studies and toxicological investigations of PFASs and other emerging pollutants compared with other soil animals or model organisms.

## 2. Toxicity of Per- and Polyfluoroalkyl Substances to *C. elegans*

### 2.1. Accumulation and Lethal Effects

PFOS bioaccumulates dose-dependently in *C. elegans* [28]. The bioaccumulation factors of PFOS, PFOA, PFBS, and PFBA in *C. elegans* were 36–1154 [28], approximately 522 [29], 2–30 [15,30], and 0.59 [31], respectively. Additionally, the 48 h LC_50_ of PFOS, PFOA, PFBS, and PFBA in *C. elegans* were 1.4–2028 μM, 4.42 μM, 793.6–1600 μM, and 2860 μM, respectively (Table 1), with a toxicity sequence of PFOS > PFOA > PFHS > PFBS > PFBA [15,28,29,30,31,32].

### 2.2. Reproductive Toxicity

Larvae at L1 were more sensitive to PFAS toxicity than those at L4, and the reproductive toxicity of PFOS in *C. elegans* was higher than that of PFOA [33], except for progeny number inhibition [34]. Furthermore, PFOS exposure impaired gonadal development in wild-type *C. elegans* L1 larvae [33], and brood amount decreased in PFOS- (concentration-dependently), PFOA-, PFBS-, and PFBA-treated L1 and L4 larvae (Table 1) [28,29,30]. Additionally, the progeny number was reduced to zero after treatment with PFOS and PFBS [15,33,35], and a prolonged generation time was observed in *C. elegans* exposed to PFOS and PFBA [31,34] (Table 2).

Reactive oxygen species (ROS) play an important role in PFOS-induced reproductive toxicity in *C. elegans* [33]. Studies have reported that ROS upregulates the expression of the meiosis-related gene *wee-1.3*, downregulates *puf-8* expression, and interferes with FB-MO-complex-related gene expression, including the upregulation of *spe-5*, *-6*, and *-17* and the downregulation of *spe-10* and *fer-1*. Additionally, ROS downregulates the expression of the sperm activation genes *swm-1* and *try-5*, resulting in reduced sperm cells, the distortion of sperm cell morphology in *him-5* larvae at L1, a reduced sperm activation rate, and a reduced offspring number caused by PFOS and PFOA. Furthermore, PFOS and PFOA affects the genes related to sperm material transport by downregulating *spe-15* and upregulating *spe-26* expression [34].

### 2.3. Cytotoxicity

Results obtained from exposing nematode strains to PFOS showed that germ cell apoptosis does not depend on the DNA damage pathway; rather, it depends on the core apoptosis, c-Jun N-terminal kinase, and p38/mitogen-activated protein kinase (MAPK) signal transduction pathways. However, germ cell cycle stagnation in *C. elegans* caused by PFOS exposure depends on the DNA damage pathway [33]. PFOS and PFBS increased germ cell apoptosis by upregulating the expression of proapoptosis-related genes, such as *egl-1* and *ced-13* [15]. Additionally, PFOS induced a significant reduction in mitochondrial content and function and key characteristics of Parkinson’s disease, such as a decreased oxygen consumption rate (oxidative phosphorylation), extracellular acidification rate, proton leakage, and nonmitochondrial oxygen consumption [36].

### 2.4. Developmental Toxicity

Exposure of *C. elegans* to PFOS, PFOA, PFBA, and PFBS inhibited growth by decreasing body length [15,28,31] and width [32]; however, only PFOS, PFBS, and GenX produced detectable developmental delays [27,36]. Additionally, PFOS-treated wild-type *C. elegans* larvae exhibited reduced life expectancy due to the expression of *daf-2* and *daf-16* [33]. Preconception exposure to PFOS and PFBS can lead to physiological dysfunction in the offspring because alterations in embryonic nutrient loading and composition delays growth rates and reduces body size in the F1 offspring of *C. elegans* [35]. Furthermore, multigenerational exposure to PFBS affected the lifespan of F4 and F5 progenies [30]. GenX in liquid media containing *C. elegans* larvae caused developmental delays, mainly the differential expression of 2624 developmentally associated genes in L4 and progeny production delays, following the sigmoidal 4PL model [27]. Lastly, PFOA, PFBS, and PFBA treatments shortened the lifespan of *C. elegans* (to approximately one-third under PFOA) [28,29,30,31].

### 2.5. Neurotoxicity

On exposure to PFOS, the GABAergic, serotonergic, and cholinergic neurons of *C. elegans* were less sensitive than the dopaminergic neurons. Additionally, the proportion of worms without neurodegeneration reduced as PFOS concentrations increased, independent of the superoxide levels [36], although neurotoxicity is closely related to oxidative damage [32]. Furthermore, decreased locomotive activity, such as head thrashes and body bending, caused by PFOS, PFOA, PFBA, or PFBS, indicates that chronic neurotoxicity and overall animal motor behavior can be transferred to *C. elegans* offspring after a few generations [30,31,32], even if the defects caused by PFOS and PFBS can be rectified in the progeny of parent nematodes exposed only once [28,29,35]. Furthermore, PFOS, PFOA, PFBS, and PFBA treatments altered chemotactic plasticity in the impaired associative learning system of nematodes [28,29,30,31]. A decreased trend index and a reduced fluorescence intensity and range of asymmetric sensory neurons caused by PFOS indicate a decreased learning and memory ability in *C. elegans*, possibly related to the selective toxicity of PFOS to specific nerve cells [32]. Lastly, Gen X induced developmental delays in worms at L2 and L3, with relatively high activity [27].

### 2.6. Oxidative Toxicity

PFOS can induce oxidative stress by promoting ROS in *C. elegans* and decreasing superoxide levels [36]. The increased ROS levels in *C. elegans* caused by PFOS or PFBS exposure are mainly achieved by the upregulated expression of antioxidant-related genes (*sod-3*, *ctl-2*, and *gst-4*) and changes in the activity of antioxidant enzymes, including the decreased activity of catalase and glutathione peroxidase [15]. Furthermore, PFOS exposure elicited a stress response and developmental effects in *C. elegans* by upregulating or downregulating the stress-response genes (*sp-16.1*, *-16.2*, *-16.48*, *-70*, *sip-1*), metallothionein proteins *mtl-1* and *mtl-2*, arsenic-induced protein-1 (*aip-1*), oxidative stress- and detoxification-associated genes *gst-4* and *skn-1*, and one development-associated gene related to the dauer formation (*daf-12*) [8]. Lastly, GenX altered the expression of detoxification-enzyme-associated genes, such as troponin C (*pat-10*) and UDP-glucuronosyltransferase (*ugt-44*) [27].

### 2.7. Metabolic Toxicity

PFOS interrupted the expression of key enzymes in the initial reaction of fatty acid synthesis in *C. elegans*, reducing the fatty acid contents in vivo, especially the unsaturated fatty acid content in L1, mainly by downregulating the gene expression of acetyl-CoA carboxylase (*acc-4*) and Δ9 desaturase (*fat-7*) [37]. Additionally, PFOS may affect the expression of other genes involved in gluconeogenesis, glycolysis, and insulin pathways related to glucose metabolism. Decreased glucose, pyruvate, and ATP contents, mainly from the downregulated gene expression of *pfk-1.1*, *gpd-2*, *pyk-1*, and *pyk-2*, and the upregulated gene expression of *hxk-2*, *pck-1*, *daf-16*, and *daf-2*, suggest a disturbance in the tricarboxylic acid cycle of *C. elegans*, which might be the cause of death following PFOS exposure [37]. Furthermore, PFOS-induced disorders of fatty acid metabolism were associated with the decreased mRNA expression of D6-desaturase genes in *C. elegans* and disrupted mitochondrial function by reducing ATP synthesis [38].

Continuous exposure to PFOA may induce obesogenic overgeneration effects, which achieved the highest level in wild-type N2 *C. elegans* in the F2 generation, offspring of F0 at the T2 generation (T1 to T3 as the offspring of F0) n, and *daf-2* mutant *C. elegans* in the F1 generation through PFOA multigenerational residual effect, derived from a complex combination of enzymes and the essential involvement of insulin signaling pathway [39]. Moreover, in wild-type *C. elegans*, the obesogenic mechanism could be attributed to the stimulation of enzymes essential for fat syntheses, such as glycerol-3-phosphate acyltransferase, acetyl-CoA carboxylase (ACC), and fatty acid synthetase (FAS), as well as the inhibition of the expression of fatty acid consumption enzymes, such as fatty acid transporter (FATP), fatty acid desaturase, acetyl-CoA synthetase, acyl-CoA oxidase, and carnitine palmitoyl transferase in fat accumulation. Furthermore, PFOA affected the critical genes of multiple signaling pathways related to the lipid metabolism, involving endogenous cannabinoids, MAPK, fatty acid degradation, glucagon, peroxisome-proliferator-activated receptor (PPAR), TGF-β, and other signaling pathways, and the transcription of *C. elegans* [24,39]. PFHS had a greater effect on the promotion sequence of lipids in wild-type N2 *C. elegans* than PFBS, and vice versa in *daf-2* mutant *C. elegans*. The mechanisms of continuous exposure differed from those under the noncontinuous exposure mode. In continuously exposed offspring, the fatty acid transporter, FATP, was inhibited in the F3 generation, and ACC and FAS were activated, resulting in lipid accumulation. Additionally, in noncontinuously exposed offspring, N-EtFOSE inhibited key enzymes of lipid metabolism, especially fatty acid β-oxidation-related enzymes, and PFBS- and PFHS-stimulated key enzymes. Lastly, the multigenerational obesogenic and residual effects of some PFASs might be related to epigenetic regulatory mechanisms, including histone modifications, such as acetylation, methylation, DNA methylation, and microRNA expression, which require further investigation [24].

### 2.8. Immunotoxicity

PFAS increased the virulence of prey; for example, by feeding on bacteria pretreated with PFOS, *bar-1*, a gene associated with multiple cancers, was upregulated in *C. elegans* [8], and PFASs could increase the virulence of *Staphylococcus aureus* by regulating the expression of genes, such as regulator saeR, α-hemolysin, and hla [40]. Additionally, PFAS increased the susceptibility of *C. elegans* to pathogens by reducing host immunity [40]. In *C. elegans* young adults, the innate immunity receptor for microbial pathogens, clec-60, was downregulated by PFOS [8]. Finally, PFASs in solution or contaminated environmental water affected the gene expression of *cpr-2*, *tag-38*, *spp-1*, *spp-5*, *clec-7*, and *clec-172* in *C. elegans*, which are involved in immune surveillance against Gram-positive bacteria, indicating increased susceptibility to certain infectious diseases and intestinal membrane permeability, resulting in the reduced survival of *C. elegans* [40].

### 2.9. Endocrine Toxicity

The endocrine-disrupting effects of PFASs were revealed by the alterations of vitellogenin (VTG) levels in *C. elegans*. PFOS and PFBS downregulated the expression of the VTG-related gene, *vit-6*, and upregulated the expression of the estrogen-receptor-related gene, *nhr-14*, suggesting that they may have potential endocrine-disrupting effects [15]. Lastly, the expression of genes encoding VTG was downregulated in worms exposed to GenX (*vit-1* to *vit-6*) [27].

## 3. Discussion

Besides the known advantages of *C. elegans*, including the compact size, relatively short life cycle, large brood size, low cost, availability of complete genetic information, relatively easy genetic manipulation, and 65% conserved human-disease-associated genes, studies using *C. elegant* do not require approval by the Institutional Animal Care and Use Committee [14]. Additionally, approximately 40% of the genes encoded by nematode proteins are homologous to human genes, and their biological processes (apoptosis, signal transduction, metabolism, and reproductive development) are highly conserved with those of mammals and humans [14]. Therefore, they can be applied in epidemiology to make up for the limitations of traditional epidemiology [41]. Moreover, they can be used to study reproductive toxicity [42], germ cell mutagenicity [43], lipid metabolism and obesity metabolic disruption [44,45], degenerative neurological diseases [46], aging process acceleration [47], and Alzheimer’s disease genetic research [48].

The reproductive system of nematodes is much simpler than that of mammals; it is completely differentiated, the structure is simple, and it is sensitive to exogenous chemicals. Reproductive toxicity in nematodes can indirectly reflect that in humans [41]. The developmental process of their germ cells is similar to that of mammals, and the location of their germ cells is relatively fixed, which is conducive to studying the developmental process of these cells [41]. According to studies, PFASs caused apoptosis in germ cells, and CED-9, CED-4, and CED-3 were vital in this process [49]. Furthermore, the proapoptosis-related gene, *egl-1* [15], encodes the expression of EGL-1, which can inhibit the activity of CED-9 and promote cellular apoptosis [50]. Additionally, the inactivation of the *p53* gene is vital in tumor formation and cellular apoptosis, and the proteins EGL-1 and CED-13, encoded by *egl-1* and *ced-13*, respectively, can induce *p53* activation [51].

PFASs with different carbon chain lengths can disrupt lipid homeostasis and have multigenerational transmissibility; short-chain alternatives have less effect on fat content than long-chain alternatives, with PFOA > PFHS > PFBS. In continuous exposure mode, the maximum fat content stimulation appeared in the F2 generation for the PFOA treatment and in F3 for the PFBS and PFHS treatments, indicating that long-term continuous exposure to PFBS and PFHS poses greater risks to the health of organisms and their offspring. Additionally, in the noncontinuous exposure mode, the residual fat accumulation effects of PFOA and PFHS were observed in generations T1 and T2, respectively. [15]. To attenuate the effects of the compound, the offspring with noncontinuous exposure recovered after exposure to compensate for the change in fat content; this recovery may be related to exposure time and concentration. Furthermore, PFASs can cause obesity in *C. elegans*, with potential developmental, neurological, or reproductive effects. This is because, in healthy *C. elegans*, the neuroendocrine control of food sensing contributes to the balance of fat accumulation [52], and dietary structure, including lipid and cholesterol contents, is a primary determinant of *C. elegans’* response to pollutants [53].

The mechanistic characterization of the effect of PFAS exposure in *C. elegans* on fat accumulation results from multiple metabolic pathways. In the transcriptome sequencing analysis, PFOA significantly affected transcription in *C. elegans*. Significant changes were observed in the multiple signaling pathways related to lipid metabolism using KEGG enrichment analysis involving endogenous cannabinoids, glucagon, MAPK, PPAR, TGF-β, and other signaling pathways. In *daf-2*-deficient *C. elegans* T3, PPAR, and TGF-β, signaling pathways were upregulated and downregulated by PFOA, leading to obesity. PPAR plays a key role in fat cell differentiation and energy storage, controlling metabolism throughout the body, and its activation increases the expression of genes that promote fatty acid storage and inhibits genes that induce lipolysis [54]. Moreover, PFOA can act as an agonist of PPARs and is known for its potency on PPAR receptors, causing lipid and steroid metabolism disorders [55,56]. Additionally, the TGF-β signaling pathway influences fat accumulation [57]. However, this pathway may only show a compensatory response when the insulin signaling pathways are turned off, supporting the important role of insulin regulation in obesity. Furthermore, alterations in selected lipid-metabolism-related signaling pathways, such as the endocannabinoid and MAPK signaling pathways, impact the organisms’ growth, development, and reproduction. Therefore, PFASs can cause obesity-producing effects in *C. elegans*, with potential developmental, neurological, or reproductive effects.

The fruit fly *Drosophila melanogaster*, the zebrafish *Danio rerio*, and the nematode worm *C. elegans* are the three traditional biomedical models [9]. These model organisms are invaluable in life science research. With three autosomal pairs and one sex chromosome, *D. melanogaster* is an excellent insect model species to investigate the effects of toxic metals, organic contaminants, and nanoparticles, attributed to the extensive experimental and genetic resources available for this species [58,59,60]. Preconception exposure of *D. melanogaster* to PFOS reduced the egg number, disordered nutrient levels, caused life developmental delays, inhibited adult weight inhibition, and prolonged female lifespan [60]. Additionally, *D. rerio*, with human genetic similarities as high as 87%, is the second-most-used animal model in medical sciences and life research, as it is an excellent vertebrate model for estimating the toxicity of chemicals with substantial advantages [61]. The toxic effects of PFASs on zebrafish have been studied for many years, revealing more extensive effects on the reproductive, developmental, cellular structure, neurologic, oxidative, metabolic, immune, and endocrine systems [62,63,64] than those achieved using *C. elegans*. Moreover, juvenile dietary exposure of *D. rerio* to PFASs could induce multigenerational behavioral effects similar to those observed in *C. elegans* [64]. Furthermore, *D. melanogaster* is an invertebrate that cannot live in water or soil/sediments, and *D. rerio* is a higher vertebrate that lives in a water environment. Although *C. elegans* is an invertebrate, it can choose either a water or soil environment as its habitat, implying that it can be used more widely for the ecological testing of water and soil/sediments than other models. Additionally, as a gynandromorph organism, *C. elegans* has high reproductive efficiency, obvious advantages in hybridization experiments, and a relatively high gene variation rate.

Soil species, especially earthworms, pot worms, springtails, and predator mites, are regularly used in toxicity assessments of soil pollutants in academic and regulatory contexts; however, the nematode *C. elegans* was the first soil-dwelling terrestrial species for which detailed ecotoxicogenomic studies (principally sequence information) were available [65]. As sufficient sequencing information from custom cDNA microarrays has become known for the earthworms, *Lumbricus rubellus* and *Eisenia fetida*, the pot worm, *Enchytraeus albidus*, and springtails, *Orchesella cincta* and *Folsomia candida*, they have been routinely utilized. Due to its good sensitivity, *E. fetida* is an earthworm species internationally accepted as the OECD standard biological indicator for monitoring soil ecological risks by direct exposure, ingestion, and passive absorption [66]. Current studies on PFASs are primarily focused on macroscopic indicators (survival, body weight, and avoidance behavior) [67,68], reproductive disruption [67], molecular responses (enzyme activity and DNA damage), metabolic responses [68], and combined toxicity with other pollutants [69]. In the toxicity evaluation of PFOS and its alternatives, such as perfluorinated butyl organic ammonium salt cationic surfactant, textile finishing agent, C4 finishing agent, and C6 finishing agent, *F. candida* showed a higher sensitivity to the toxicity of the alternatives than *Gobiocypris rarus*, even when the cultivation time was longer [62,70,71]. Nevertheless, the indicator screening, sensitivity, and adept systems of different soil species should be compared.

**Figure 1:**
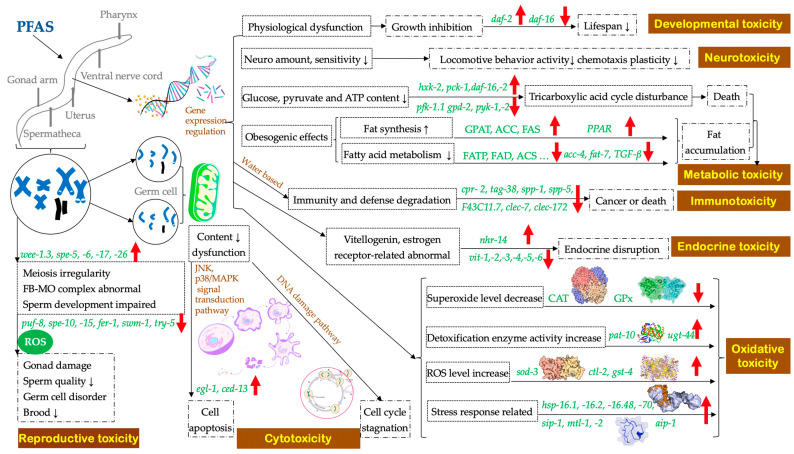
General toxicological mechanisms of PFASs on *C. elegans*.

The toxicity of PFASs has not been thoroughly investigated because only five of them have been studied. The general toxicity mechanisms of the commonly detected PFASs in *C. elegans* were similar (Figure 1), with the toxicity order as PFOS > PFOA > PFBS > PFBA.

GenX induced physiological effects, including developmental delay, behavior and locomotive effects, and transcriptional effects; however, further comparisons between GenX and the other four PFASs are needed. Additionally, molecular indices have higher sensitivity and specificity than physiological and chemical indices [15]. Therefore, further studies on the neurotoxic effects of PFAS relating to molecular mechanisms and gene expression are necessary. Furthermore, as a low-cost and highly effective model organism, *C. elegans* should be used in more toxicity tests of other compounds with different carbon chain lengths in the huge PFAS family, including perfluorodecanoic acid, perfluopentanoic acid [72], PFHxA, perfluoropentanesulfonic acid (PFPeS), perfluorohexyl sulfonate (PFHxS), perfluorononanoic acid, perfluorooctane sulfonamide (PFOSA), perfluorododecanoic acid (PFDoA), perfluorodecanoic acid, perfluorodecanoic acid, perfluoroheptanoic acid [67], ammonium perfluorooctanoate, perfluoropropionic acid, fluorotelomer carboxylic acids, fluorotelomer alcohols, perfluoroundecanoic acid, perfluoroethylcyclohexane sulfonate [2], and other short-chain analogs, such as chlorinated polyfluoroalkyl ether sulfonic acids and sodium p-perfluorous nonenoxybenzenesulfonate, widely used as alternatives to PFOS and PFOA [6].

Unlike the known effect of PFASs on the male reproductive system, such as malformation of reproductive organs or gonads and decreased sperm quality, little is known about the impact of PFASs on female germ cell development in *C. elegans.* In related studies, PFAS exposure weakened oocyte quality, arrested oocyte maturation, increased ROS levels and DNA damage, as well as caused failed sperm binding and fertilization and oxidative-stress-induced apoptosis of porcine oocytes [73].

## 4. Conclusions

Based on the bioaccumulation level and LC_50_ of the reported PFASs in *C. elegans*, the toxicity sequence is as follows: PFOS > PFOA > PFHS > PFBS > PFBA. However, investigations into the toxic effects of other PFASs and their substitutes remain insufficient. PFASs can induce toxic effects in different systems, especially in the metabolic, reproductive, and neurologic systems, and can cause transgenerational effects on the organism’s development. However, their toxicity on the genes and immune systems of *C. elegans* has not received detailed coverage. Therefore, studies on the neurotoxic effects of PFASs on molecular mechanisms and gene expression in *C. elegans* are required for further guidance in treating human diseases. *C. elegans* has traditional and unique advantages over other traditional biomedical models and soil fauna in academic research, besides being relevant to human epidemiological research on diseases such as obesity, Alzheimer’s disease, and Parkinson’s disease. Lastly, further studies on *C. elegans* should be conducted to elucidate the toxicity mechanisms of emerging pollutants.

## Figures and Tables

**Table 1:** LC_50_ values of target PFASs and some substitutes in *C. elegans*.

Pollutant	Indicator	Value	Reference
PFOS	48 h	1.4 μM (0.699 mg/L)	[15]
PFBS		793.6 μM (238.82 mg/L)	
PFOS	48 h	3.15 μM (1.575 mg/L)	[28]
PFOA	48 h	4.42 μM (1.83 mg/L)	[29]
PFBS	48 h	1.60 mM (481.5 mg/L)	[30]
PFBA	48 h	2.86 mM (612.15 mg/L)	[31]
PFOS	24 h	3.484 mM (1738.76 mg/L)	[32]
	48 h	2.028 mM (1012.11 mg/L)	
	72 h	0.842 mM (420.22 mg/L)	

PFOS, perfluorooctane sulfonic acid; PFOA, perfluorooctanoic acid; PFBA, perfluorobutanoic acid; PFBS, perfluorobutane sulfonate.

**Table 2:** Macroscopic characteristics of the toxic effects of PFASs on reproductivity and the development of *C. elegans*.

Effect	Impairment Detail	Pollutant	Reference
Gonad damage	Germ cell arrangement disorder	PFOS	[33]
	Cell spacing increase		
	Gonad size decrease		
	Gonad cell number decrease		
Brood inhibition	Brood amount/size (total egg number) decrease	PFOS, PFBS, PFOA, PFBA	[28,29,30]
	Progeny number (hatched offspring number) decrease	PFOA, PFOS, PFBS	[15,33,34]
	Generation time prolongation	PFOS, PFBA	[31,34]
Growth inhibition	Body length/area decrease	PFOS, PFOA, PFBA, PFBS	[15,28,31,33]
	Body width decrease	PFOS	[32]
	Developmental delay	PFOS, PFBS, GenX	[27,33]
	Progeny production delay	GenX	[27]
Life expectancy inhibition		PFOS, PFOA, PFBA, PFBS	[28,29,30,31,33]

GenX, hexafluoropropylene oxide dimer acid.

## Data Availability

The data presented in this study are available on request from the corresponding author.

## Supplementary Materials

The following supporting information can be downloaded at: https://www.mdpi.com/article/10.3390/toxics11070593/s1, Table S1: General description of the methodology and scope of work in referred *C. elegans* studies.

## References

[1] Gockener B., Fliedner A., Rudel H., Badry A., Koschorreck J. (2022). Long-term trends of per- and polyfluoroalkyl substances (PFAS) in suspended particular matter from German rivers using the direct total oxidizable precursor (dTOP) assay. Environ. Sci. Technol..

[2] Stahl T., Mattern D., Brunn H. (2011). Toxicology of perfluorinated compounds. Environ. Sci. Eur..

[3] Huang K., Li Y., Bu D., Fu J., Wang M., Zhou W., Gu L., Fu Y., Cong Z., Hu B. (2022). Trophic Magnification of Short-Chain Per- and Polyfluoroalkyl Substances in a Terrestrial Food Chain from the Tibetan Plateau. Environ. Sci. Technol. Lett..

[4] Kurwadkar S., Dane J., Kanel S.R., Nadagouda M.N., Cawdrey R.W., Ambade B., Struckhoff G.C., Wilkin R. (2022). Per- and polyfluoroalkyl substances in water and wastewater: A critical review of their global occurrence and distribution. Sci. Total Environ..

[5] Rashid F., Dubinkina V., Ahmad S., Maslov S., Irudayaraj J.M.K. (2023). Gut Microbiome-Host Metabolome Homeostasis upon Exposure to PFOS and GenX in Male Mice. Toxics.

[6] Ma T., Ye C., Wang T., Li X., Luo Y. (2022). Toxicity of Per- and Polyfluoroalkyl Substances to Aquatic Invertebrates, Planktons, and Microorganisms. Int. J. Environ. Res. Public Health.

[7] Putten V.D., Wim H., Bardgett D.R. (2014). Belowground biodiversity and ecosystem functioning. Nature.

[8] Yin W.Y. (2000). Soil Animal in China.

[9] Douglas A.E. (2019). Simple animal models for microbiome research. Nat. Rev. Microbiol..

[10] Van Den Hoogen J., Geisen S., Wall D.H., Wardle D.A., Traun-Spurger W., De Goede R.G.M., Adams B.J., Ahmad W., Ferris H.L., Bardgett R.D. (2020). A global database of soil nematode abundance and functional group composition. Sci. Data.

[11] Brazova T., Kovacik P., Matouskova M., Oros M. (2022). Nematodes as soil stress indicators for polycyclic aromatic hydrocarbons: A review. Helminthologia.

[12] Wang X., Nielsen U.N., Yang X., Zhang L., Zhou X., Du G., Li G., Chen S., Xiao S. (2018). Grazing induces direct and indirect shrub effects on soil nematode communities. Soil Biol. Biochem..

[13] Stylianou M., Björnsdotter M.K., Olsson P.-E., Jogsten I.E., Jass J. (2019). Distinct transcriptional response of *Caenorhabditis elegans* to different exposure routes of perfluorooctane sulfonic acid. Environ. Res..

[14] Yue Y., Li S., Shen P., Park Y. (2021). *Caenorhabditis elegans* as a model for obesity research. Curr. Res. Food Sci..

[15] Chen F.J. (2017). Toxicity of Perfluorobutane Sulfonate as a Substitute for Perfluorooctane Sulfonate on *Caenorhabditis elegans*. Master’s Thesis.

[16] Lakowski B., Hekimi S. (1996). Determination of life-span in *Caenorhabditis elegans* by four clock genes. Science.

[17] Gubert P., Gubert G., Oliveira R.C.D., Fernandes I.C.O., Bezerra I.C., de Ramos B., de Lima M.F., Rodrigues D.T., de Cruz A.F.N., Pereira E.C. (2023). *Caenorhabditis elegans* as a Prediction Platform for Nanotechnology-Based Strategies: Insights on Analytical Challenges. Toxics.

[18] Hitchcock D.R., Black M.C., Williams P.L. (1997). Investigations into using the nematode *Caenorhabditis elegans* for municipal and industrial wastewater toxicity testing. Arch. Environ. Contam. Toxicol..

[19] Dietrich N., Tan C.-H., Cubillas C., Earley B.J., Kornfeld K. (2016). Insights into zinc and cadmium biology in the nematode *Caenorhabditis elegans*. Arch. Biochem. Biophys..

[20] Meyer D., Williams P.L. (2014). Toxicity Testing of Neurotoxic Pesticides in *Caenorhabditis elegans*. J. Toxicol. Environ. Health Part B.

[21] Zhao Y.L., Chen J.Y., Wang R., Pu X.X., Wang D.Y. (2023). A review of transgenerational and multigenerational toxicology in the in vivo model animal *Caenorhabditis elegans*. J. Appl. Toxicol..

[22] Haimbaugh A., Wu C.-C., Akemann C., Meyer D.N., Connell M., Abdi M., Khalaf A., Johnson D., Baker T.R. (2022). Multi- and Transgenerational Effects of Developmental Exposure to Environmental Levels of PFAS and PFAS Mixture in Zebrafish (*Danio rerio*). Toxics.

[23] Wang Z., Cousins I.T., Scheringer M., Hungerbühler K. (2013). Fluorinated alternatives to long-chain perfluoroalkyl carboxylic acids (PFCAs), perfluoroalkane sulfonic acids (PFSAs) and their potential precursors. Environ. Int..

[24] Li Z. (2020). Multi-Generational Effects of Perfluorinated Compounds (PFCs) on Lipid Metabolism of *C. elegans* and Its Potential Mechanism. Master’s Thesis.

[25] Sun B., Liu M., Tang L., Hu C., Chen L. (2021). Probiotic supplementation mitigates the developmental toxicity of perfluorobutanesulfonate in zebrafish larvae. Sci. Total Environ..

[26] Ma T., Wu P., Ding Z., Wang T., Luo Y. (2022). Pet cats, the better sentinels for indoor organic pollutants. Front. Environ. Sci..

[27] Feng Z., McLamb F., Vu J.P., Gong S., Gersberg R.M., Bozinovic G. (2022). Physiological and transcriptomic effects of hexafluoropropylene oxide dimer acid in *Caenorhabditis elegans* during development. Ecotoxicol. Environ. Saf..

[28] Chowdhury M.I., Sana T., Panneerselvan L., Sivarama A.K., Megharaj M. (2022). Perfluorooctane sulfonate (PFOS) induces several behavioural defects in *Caenorhabditis elegans* that can also be transferred to the next generations. Chemosphere.

[29] Sana T., Chowdhury M.I., Logeshwaran P., Dharmarajan R., Megharaj M. (2021). Perfluorooctanoic acid (PFOA) induces behavioural, reproductive and developmental toxicological impacts in *Caenorhabditis elegans* at concentrations relevant to the contaminated areas. Environ. Adv..

[30] Chowdhury M.I., Sana T., Panneerselvan L., Dharmarajan R., Megharaj M. (2021). Acute Toxicity and Transgenerational Effects of Perfluorobutane Sulfonate on *Caenorhabditis elegans*. Environ. Toxicol. Chem..

[31] Sana T., Chowdhury M.I., Logeshwaran P., Megharaj M. (2023). Behavioural, developmental and reproductive toxicological impacts of perfluorobutanoic acid (PFBA) in *Caenorhabditis elegans*. Environ. Chall..

[32] Chen N. (2014). Neurotoxicity of Chronic Exposure to Perfluorooctane Sulfonate and Its Mechanisms, In Vitro and In Vivo. Master’s Thesis.

[33] Guo X.Y. (2014). The Toxicity of Growth and Germ Line Development Induced by Perfluorooctane Sulfonates in Caenorhabditis elegans. Doctor’s Thesis.

[34] Jian Z.H. (2017). The Male Reproductive Toxicity of Perfluorooctane Sulfonates and Perfluorooctanoic Acid on *C. elegans*. Master’s Thesis.

[35] Yue Y., Li S., Qian Z., Pereira R.F., Lee J., Doherty J.J., Zhang Z., Peng Y., Clark J.M., Timme-Laragy A.R. (2020). Perfluorooctanesulfonic acid (PFOS) and perfluorobutanesulfonic acid (PFBS) impaired reproduction and altered offspring physiological functions in *Caenorhabditis elegans*. Food Chem. Toxicol..

[36] Sammi S.R., Foguth R.M., Nieves C.S., De Perre C., Wipf P., McMurray C.T., Lee L.S., Cannon J.R. (2019). Perfluorooctane Sulfonate (PFOS) Produces Dopaminergic Neuropathology in *Caenorhabditis elegans*. Toxicol. Sci..

[37] Wei C.Y. (2018). Effects of PFOS on Lipid and Glucose Metabolism in *Caenorhabditis elegans*. Master’s Thesis.

[38] Wei C., Zhou Z., Wang L., Huang Z., Liang Y., Zhang J. (2021). Perfluorooctane sulfonate (PFOS) disturbs fatty acid metabolism in *Caenorhabditis elegans*: Evidence from chemical analysis and molecular mechanism exploration. Chemosphere.

[39] Li Z., Yu Z., Gao P., Yin D. (2020). Multigenerational effects of perfluorooctanoic acid on lipid metabolism of *Caenorhabditis elegans* and its potential mechanism. Sci. Total Environ..

[40] Mangu J.C.K., Stylianou M., Olsson P.-E., Jass J. (2022). Per- and polyfluoroalkyl substances enhance Staphylococcus aureus pathogenicity and impair host immune response. Environ. Pollut..

[41] Hanssen L., Röllin H., Odland J.Ø., Moe M.K., Sandanger T.M. (2010). Perfluorinated compounds in maternal serum and cord blood from selected areas of South Africa: Results of a pilot study. J. Environ. Monit..

[42] Liu X., Ge P., Lu Z., Yang R., Liu Z., Zhao F., Chen M. (2022). Reproductive toxicity and underlying mechanisms of fine particulate matter (PM2.5) on *Caenorhabditis elegans* in different seasons. Ecotoxicol. Environ. Saf..

[43] Feng Y., Cao Z., Xu A., Du H. (2023). Evaluation of toxicity and mutagenicity of oxaliplatin on germ cells in an alternative in vivo model *Caenorhabditis elegans*. Food Chem. Toxicol..

[44] Lin T.A., Huang C.W., Wei C.C. (2022). Early-life perfluorooctanoic acid (PFOA) and perfluorooctane sulfonic acid (PFOS) exposure cause obesity by disrupting fatty acids metabolism and enhancing triglyceride synthesis in *Caenorhabditis elegans*. Aqua. Toxicol..

[45] Haerkens F., Kikken C., Kirkels L., van Amstel M., Wouters W., van Doornmalen E., Francke C., Hughes S. (2022). A new use for old drugs: Identifying compounds with an anti-obesity effect using a high through-put semi-automated *Caenorhabditis elegans* screening platform. Heliyon.

[46] Lee Y., Choi S., Kim K.W. (2023). Dithianon exposure induces dopaminergic neurotoxicity in *Caenorhabditis elegans*. Ecotoxicol. Environ. Saf..

[47] How C.M., Li S.W., Liao V.H.C. (2018). Chronic exposure to triadimenol at environmentally relevant concentration adversely affects aging biomarkers in *Caenorhabditis elegans* associated with insulin/IGF-1 signaling pathway. Sci. Total Environ..

[48] Mukherjee S., Russell J.C., Carr D.T., Burgess J.D., Younkin M.A., Serie D., Boehme K.L., Kauwe J.S.K., Naj A.C., Fardo D.W. (2017). Systems biology approach to late-onset Alzheimer’s disease genome-wide association study identifies novel candidate genes validated using brain expression data and *Caenorhabditis elegans* experiments. Alzheimer’s Dement..

[49] Yan N., Chai J., Lee E.S., Gu L., Liu Q., He J., Wu J.-W., Kokel D., Li H., Hao Q. (2005). Structure of the CED-4–CED-9 complex provides insights into programmed cell death in *Caenorhabditis elegans*. Nature.

[50] Conradt B., Horvitz A.H.R. (1998). The Protein EGL-1 Is Required for Programmed Cell Death and Interacts with the Bcl-2–like Protein CED-9. Cell.

[51] Schumacher B., Schertel C., Wittenburg N., Tuck S., Mitani S., Gartner A., Conradt B., Shaham S. (2005). *C. elegans* ced-13 can promote apoptosis and is induced in response to DNA damage. Cell Death Differ..

[52] Srinivasan S. (2020). Neuroendocrine control of lipid metabolism: Lessons from *C. elegans*. J. Neurogenet..

[53] Crawford N., Martell M., Nielsen T., Khalil B., Imtiaz F., Nguidjo E., Newell-Caito J.L., Bornhorst J., Schwerdtle T., Caito S.W. (2021). Methylmercury-Induced Metabolic Alterations in *Caenorhabditis elegans* Are Diet-Dependent. Toxics.

[54] Berger J.P., Akiyama T.E., Meinke P.T. (2005). PPARs: Therapeutic targets for metabolic disease. Trends Pharmacol. Sci..

[55] Maradonna F., Carnevali O. (2018). Lipid Metabolism Alteration by Endocrine Disruptors in Animal Models: An Overview. Front. Endocrinol..

[56] Dewitt J.C. (2015). Toxicological Effects of Perfluoroalkyl and Polyfluoroalkyl Substances.

[57] Sze J.Y., Victor M., Loer C., Shi Y., Ruvkun G. (2000). Food and metabolic signalling defects in a *Caenorhabditis elegans* serotonin-synthesis mutant. Nature.

[58] Slobodian M.R., Petahtegoose J.D., Wallis A.L., Levesque D.C., Merritt T.J.S. (2021). The Effects of Essential and Non-Essential Metal Toxicity in the *Drosophila melanogaster* Insect Model: A Review. Toxics.

[59] Wang Z., Zhang L., Wang X. (2023). Molecular toxicity and defense mechanisms induced by silver nanoparticles in *Drosophila melanogaster*. J. Environ. Sci..

[60] Kim J.H., Barbagallo B., Annunziato K., Farias-Pereira R., Doherty J.J., Lee J., Zina J., Tindal C., McVey C., Aresco R. (2021). Maternal preconception PFOS exposure of *Drosophila melanogaster* alters reproductive capacity, development, morphology and nutrient regulation. Food Chem. Toxicol..

[61] Shen C., Zuo Z. (2020). Zebrafish (*Danio rerio*) as an excellent vertebrate model for the development, reproductive, cardiovascular, and neural and ocular development toxicity study of hazardous chemicals. Environ. Sci. Pollut. Res..

[62] Ma T., Wu P., Wang L., Li Q., Li X., Luo Y. (2023). Toxicity of per- and polyfluoroalkyl substances to aquatic vertebrates. Front. Environ. Sci..

[63] Fey M.E., Goodrum P.E., Razavi N.R., Whipps C.M., Fernando S., Anderson J.K. (2022). Is Mixtures’ Additivity Supported by Empirical Data? A Case Study of Developmental Toxicity of PFOS and 6:2 FTS in Wildtype Zebrafish Embryos. Toxics.

[64] Rericha Y., Truong L., Leong C., Cao D., Field J.A., Tanguay R.L. (2022). Dietary Perfluorohexanoic Acid (PFHxA) Exposures in Juvenile Zebrafish Produce Subtle Behavioral Effects across Generations. Toxics.

[65] Spurgeon D.J., Morgan A.J., Kille P. (2008). Current research in soil invertebrate ecotoxicogenomics. Adv. Exp. Biol..

[66] Organization for Economic Cooperation and Development (1984). Acute Toxicity Tests. Guideline for Testing Chemicals.

[67] Melo T.M., Schauerte M., Bluhm A., Slany M., Paller M., Bolan N., Bosch J., Fritzsche A., Rinklebe J. (2022). Ecotoxicological effects of per- and polyfluoroalkyl substances (PFAS) and of a new PFAS adsorbing organoclay to immobilize PFAS in soils on earthworms and plants. J. Hazard. Mater..

[68] Cai Y., Wang Q., Zhou B., Yuan R., Wang F., Chen Z., Chen H. (2021). A review of responses of terrestrial organisms to perfluorinated compounds. Sci. Total Environ..

[69] Wang Z., Shi Y., Zhang Z., Qi F., Xue W., Tian D., Teng S. (2022). Assessing joint toxic effects of arsenate and perfluorooctane sulfonate on earthworm by combining integrated biomarker response and mixture toxicity indices. Ecolo. Indic..

[70] Zhang C., Zhang X., Xu Y., Qiao M. (2012). Ecotoxicity of Alternatives of Typical Perfluorooctane Sulfonate (PFOS) to Springtails. Asian J. Ecotoxicol..

[71] Zhang X., Zhang C., Wang G., Qiao M., Zhu Y. (2012). Ecotoxicity of Perfluorooctane Sulfonate (PFOS) to Springtails in Soils. Asian J. Ecotoxicol..

[72] Groffen T., Prinsen E., Stoffels O.A.D., Maas L., Vincke P., Lasters R., Eens M., Bervoets L. (2023). PFAS accumulation in several terrestrial plant and invertebrate species reveals species-specific differences. Environ. Sci. Pollut. Res..

[73] Chen J.Y., Miao Y.L., Gao Q., Cui Z.K., Xiong B. (2021). Exposure to perfluorooctane sulfonate in vitro perturbs the quality of porcine oocytes via induction of apoptosis. Environ. Pollut..

