# Supplementary material for: Toxicity of Per- and Polyfluoroalkyl Substances to Nematodes
Source: Toxics. 2023 Jul 7;11(7):593. doi: 10.3390/toxics11070593 (PMC10385831; doi:10.3390/toxics11070593)
Supplement: Supplementary file 1 [file toxics-11-00593-s001.zip › toxics-2371518-supplementary.pdf]

Table S1 General description of the methodology and scope of work in referred *C. elegans* studies.

Effect	Life stage	Exposure mode	Pollutant	Type	Ref
Lethal, body length, reproduction behavior, bioaccumulation, in vivo apoptosis, in vivo ROS, in vivo antioxidant oxidase, expression of vitellinogen related genes, estrogen receptor-related genes, pro-apoptosis-related genes, antioxidant-related genes	L1 to L4	Agar growth medium	PFOS, PFBS	Wild-type Bristol N2 strain	[15]
Fat storage and lipid metabolism	F0 to F3	Agar growth medium	PFOA	Wild-type Bristol N2 strain; *daf-2* mutant	[24]
Behavior and locomotion effects, developmental delay, gene expression changes in reproduction, neuro system, detoxification-enzyme-associated genes, and VTG related	L1 to L4	Agar growth medium	HFPO-DA	Wild-type Bristol N2 strain	[27]
Lethal, bioaccumulation, body growth, reproduction behavior, locomotion behavior, chemotaxis assay, lifespan, transgenerational reproduction, locomotion, and lifespan behavior	L1 to L4	Standard agar growth medium	PFOS	Wild-type Bristol N2 strain	[28]
Lethal, bioaccumulation, reproduction behavior, growth, locomotion behavior, developmental defects, chemotaxis plasticity, and lifespan	L4	Agar growth medium	PFOA	Wild-type Bristol N2 strain	[29]
Lethal, bioaccumulation, generation time, reproduction behavior, life span, locomotion behavior, body growth, chemotaxis plasticity, and transgenerational effect	L1 to L4	Standard agar growth medium	PFBS	Wild-type Bristol N2 strain	[30]
Lethal, bioaccumulation, reproduction behavior, locomotion behavior, chemotaxis plasticity, growth, and lifespan	L4	Agar growth medium	PFBA	Wild-type Bristol N2 strain	[31]
Lethal, body growth, locomotion behavior, chemotaxis, and neuron visual analysis	L1	Agar growth medium	PFOS	Wild-type Bristol N2 strain; OH2871 (gcy-5::GPF), OH10819 (unc-17::GFP), BZ555(pDAT::GFP)	[32]
Body growth, lethal, lifespan, reproduction behavior, including gonad development and male ratio, cytotoxicity on germ cells, expression of genes related to reduced body surface area, shortened lifespan, germ cell apoptosis, and cell cycle arrest	L1 to L4	Liquid medium	PFOS	Wild-type Bristol N2 strain	[33]

Reproduction behavior, lethal, lifespan reproductive ability including sperm number, sperm cell morphology and size, and sperm activation	L1 to L4	Agar growth medium	PFOS, PFOA	Wild-type Bristol N2 strain; *him-5* (*e1490*), *fog-2* (*q71*), *spe-4* (*hc78*), *spe-4* (*hc93*), *spe-6* (*hc163*), *spe-10* (*hc104*), *fer-1* (*hc1*), *swm-1* (*me66*)	[34]
Reproduction, pumping rate, development inhibition, locomotive activity change, body size, growth rate, and bioaccumulation	L1 to L4	Liquid medium	PFOS, PFBS	Wild-type Bristol N2 strain; *DH1033* (*sqt-1* (*sc103*) *II*; *bIs1 X*)	[35]
Bioaccumulation, neurodegeneration, expression of neuro system and antioxidant-related genes, cytotoxicity effects, cell mitosis, and motor behavior	L1	Liquid medium	PFOS, PFOA	wild-type Bristol N2 strain; BZ555 (egIs1[dat-1p::GFP]), CZ1632 (juIs76 [unc-25p::GFP + lin-15(+)]), GR1366 (mgIs42 [tph-1::GFP + rol-6(su1006)]), LX929 (vsIs[unv-17::GFP])	[36]
Transgenerational fat storage, lipid metabolism, and gene expression profiles	F0 to F3	Agar growth medium	PFOA	Wild-type Bristol N2 strain; *daf-2* mutant	[39]
Immune response gene expression, bioaccumulation, and lethal	L1 to L4	Agar growth medium	PFOS, PFHxS	Wild-type Bristol N2 strain	[40]
